# Transgenic mice overexpressing human TNF-α experience early onset spontaneous intervertebral disc herniation in the absence of overt degeneration

**DOI:** 10.1038/s41419-018-1246-x

**Published:** 2018-12-18

**Authors:** Deborah J. Gorth, Irving M. Shapiro, Makarand V. Risbud

**Affiliations:** Department of Orthopaedic Surgery and Graduate Program in Cell Biology and Regenerative Medicine, Thomas Jefferson University, Philadelphia, PA USA

## Abstract

There is a well-established link between cytokine expression and the progression of intervertebral disc degeneration. Among these cytokines, interleukin-1β (IL-1β) and tumor necrosis factor-α (TNF-α) are the most commonly studied. To investigate whether systemic hTNF-α overexpression affects intervertebral disc health, we studied the spine phenotype of Tg197 mice, a widely used hTNF-α transgenic line. These mice were studied at 12–16 weeks of age using comprehensive histochemical and immunohistological analysis of the spinal motion segment. Micro-CT analysis was performed to quantify vertebral trabecular bone architecture. The Tg197 mice evidenced spontaneous annular tears and herniation with increased vascularity in subchondral bone and significant immune cell infiltration. The full-thickness annular tear without nucleus pulposus (NP) extrusion resulted in neutrophil, macrophage, and mast cell infiltration into the disc, whereas the disc with full-thickness tear and pronounced NP herniation showed additional presence of CD4+ and CD8+ T cells. While the observed defects involved failure of the annular, endplate, and vertebral junction, there were no obvious alterations in the collagen or aggrecan content in the NP and annulus fibrosus or the maturity of collagen fibers in Tg197 mice. Despite elevated systemic inflammation and pronounced loss of trabecular bone in the vertebrae, intact Tg197 discs were healthy and showed an increase in NP cell number. The NP cells in intact discs preserved expression of phenotypic markers: CAIII, Glut1, and Krt19. In conclusion, elevated systemic TNF-α increases the susceptibility of mice to spontaneous disc herniation and possibly radiculopathy, without adversely affecting intact intervertebral disc health.

## Introduction

Low back pain (LBP) is a profoundly debilitating and increasingly prevalent condition with a huge societal cost^1^. LBP is currently the leading cause of disability worldwide; a recent study of the US and global population ranked LBP as the first, and neck pain as the fourth condition for years lived with disability^2,3^. The health of the intervertebral disc is intricately linked with LBP^4^. Patients with severely degenerated discs are 3.2 times more likely to suffer from LBP^5^.

The disc comprises an inner gelatinous glycosaminoglycan-rich nucleus pulposus (NP) surrounded circumferentially by an organized fibrocartilaginous annulus fibrosus (AF) and inferiorly and superiorly by cartilaginous endplates (CEP). Disc degeneration is characterized by increased fibrosis and decreased proteoglycan content in the NP leading to reduced ability of the tissue to bind and retain water, thereby compromising the mechanical properties of the motion segment^6–8^. There is also evidence of increased cell death and a transition from notochordal cells to cells that exhibit the characteristics of hypertrophic chondrocytes^7,9^. Pro-inflammatory cytokine expression is correlated with the severity of disc degeneration^10^. Several studies have shown that degenerated discs exhibit increased expression of chemokines and inflammatory cytokines, and there is evidence of immune cell infiltration^11,12^. Inflammatory cytokines are produced both by NP and AF cells as well as by infiltrating immune cells in herniated discs. Through activation of matrix metalloproteinases (MMPs) and other proteases, cytokines cause extracellular matrix breakdown and enhance recruitment of immune cells thereby perpetuating and promoting the inflammatory environment^13–15^. Among these cytokines, interleukin-1β (IL-1β) and tumor necrosis factor-α (TNF-α) are the most commonly studied. In addition to its primary physiological role, the regulation of immune cell function, TNF-α paradoxically induces both apoptotic cell death as well as cell proliferation and differentiation^16–18^. TNF-α dysfunction has been implicated in the pathogenesis of numerous disorders, including rheumatoid arthritis, asthma, septic shock, irritable bowel syndrome, and most relevant to the present study, intervertebral disc disease^19,20^. Anti-TNF-α therapies are currently being investigated for the treatment of disc degeneration, albeit with mixed results^21–23^.

TNF-α is elevated in numerous systemic inflammatory conditions related to disc health. High levels of circulating cytokines including TNF-α are seen in patients suffering radiculopathy following disc herniation^24,25^. Olmarker and colleagues have shown that TNF-α recapitulates effects of herniated NP tissue on dorsal root ganglion apoptosis in a rat model of disc herniation^26^. Similarly, a recent study by Lai et al. has demonstrated increased pain behavior when TNF-α was injected in a rat model of disc puncture^27^. Additionally, elevated levels of TNF-α in individuals with high body mass index correlates with both disc degeneration and LBP^28^. Increased TNF-α is also seen in diabetic patients, a systemic inflammatory condition correlated to disc disease^29,30^. Likewise, inflammation in the neighboring vertebrae is associated with symptomatic disc disease. Modic Type 1 changes are strongly correlated with LBP and are indicative of bone edema linked to inflammation^31,32^. Importantly, there is a link between marrow edema and levels of circulating TNF-α^33^. In summary, despite the association between inflammation and disc disease, particularly elevated TNF-α levels, the causative relationship between this cytokine and disc disease is not firmly established.

A human TNF-α-overexpressing transgenic mouse line (Tg197) is widely used to investigate the role of systemic hTNF-α overexpression in inflammation-driven pathologies^34–36^. These mice exhibit early-onset polyarthritis that affects major arthrodial joints; this is characterized by robust inflammation and structural degradation of synovium, articular cartilage, and bone. To examine the contribution of elevated systemic TNF-α levels and vertebral bone inflammation to disc herniation and degeneration, we characterized the spinal phenotype of Tg197 mice. Our studies show that chronic inflammation due to elevated systemic TNF-α promotes annular tears, herniation, and consequently immune cell infiltration in the discs. Surprisingly, unaffected discs maintained their structural integrity with minimal changes in the NP and AF tissues.

## Results

### Tg197 mice show a higher incidence of disc herniations characterized by immune cell infiltration

**Fig. 1:**
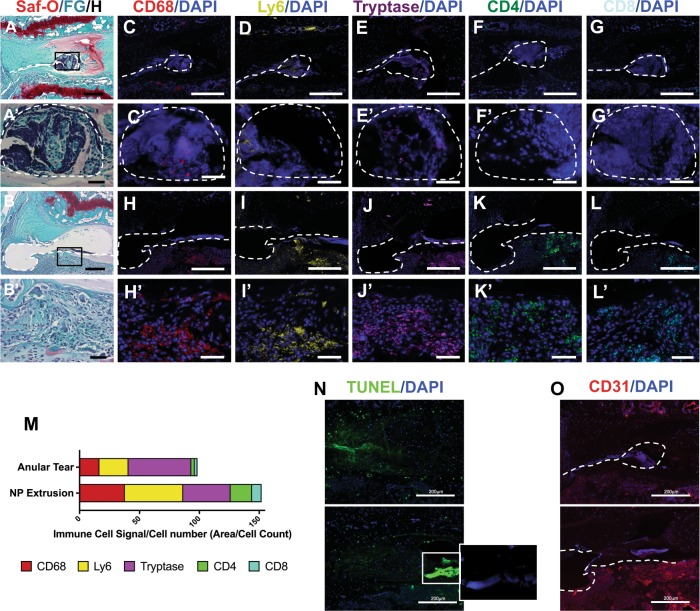
Immune cell response to spontaneous herniation in Tg197 caudal discs. **a**, **b**’ Left column shows Safranin O/Fast Green/Hematoxylin staining of spontaneous caudal herniation. Inset box (**a**, **b**) shows the location of the higher magnification image beneath (**a**’, **b**’). Each row shows staining of successive sections of the same region, with white dashed outlines for positional context. Immunofluorescence staining of the annular tear shows CD68-positive cells (macrophages) (**c**, **c**’), Ly6-positive cells (neutrophils) (**d**, **d**’), and tryptase-positive cells (mast cells) (**e**, **e**’), Note: these sections were negative for CD4 and CD8, T cell markers (**f**, **g**’). The nucleus pulposus extrusion shows a large immune cell infiltration positive for all macrophage (**h**, **h**’), neutrophil (**i**, **i**’), mast cell (**j**, **j**’), and T cell markers, including both CD4 and CD8 (**k**, **l**’) (first and third row, scale bars = 200 μm; second and fourth row, scale bars = 50 μm). **m** Quantitation of the immune signal of each marker. **n** TUNEL staining of herniated disc sections. Insert box showing DAPI signal beneath cluster of cells displaying high intensity TUNEL signal on bottom image. **o** CD31 staining showing increased vascularity in the subchondral bony endplate of herniated discs

Our analysis of Tg197 mice at 12–16 weeks showed that three of the ten animals exhibited spontaneous disc herniation at one of the three caudal levels interrogated (Fig. 1a, b). In contrast, none of the discs in wild-type (WT) mice showed signs of annular defects or herniation. In one instance, the annular tear appeared as a cleft across the entire width of the AF, stretching from the endplate–AF junction to the NP (Fig. 1a). There was a large population of cells that infiltrated into the aggrecan matrix of the NP; the remnant NP cells did not display the vacuolar morphology of healthy discs (Fig. 1a, a’). The other herniated disc also exhibited a full thickness cleft; however, the NP extracellular matrix and cells had extruded outside the disc space and were surrounded by cells (Fig. 1b). The cell response at the site of herniation disrupted the integrity of the growth plate adjacent to the endplate–AF junction. To verify the identity of the infiltrating cells and to further characterize the nature of the response, we performed immunofluorescence staining using a panel of well-defined immune cell markers.

In the NP compartment of discs exhibiting annular tear only, cells stained positive for CD68: a macrophage marker, Ly6: a neutrophil marker, and Tryptase: a mast cell marker (Fig. 1c–e’). However, staining for both CD4 and CD8, T cell markers, was limited and confined to few positive cells in the vasculature of the neighboring vertebral body (Fig. 1f, g’). Tryptase-positive cells were most often centrally located in the NP (Fig. 1e, e’), followed by CD68-positive cells (Fig. 1c, c’); the Ly6-positive cells were clustered between the CD68+ cells and the endplate–AF end of the cleft (Fig. 1d, d’). The lack of T cell staining in the disc with annular tears suggested this to be an acute event (Fig. 1f, g’). In contrast, in addition to other immune cell types (Fig. 1h–j’), the disc with both AF tear and NP extrusion stained positive for CD4 and CD8 (Fig. 1k, l’). The staining for all the cell types was most pronounced in the vertebral body adjacent to the endplate–AF junction through which the AF cleft propagated and did not show any specific pattern. To gain an overall understanding of the nature of this immune response, the staining for each marker was quantified (Fig. 1m). It is interesting to note that herniation lead to extensive cell death in the disc compartment (Fig. 1n). In addition, both types of herniated discs showed elevated CD31 staining in the subchondral bony plate, suggesting increased vascularity (Fig. 1o).

### Tg197 mice do not show evidence of early-onset disc degeneration in intact discs

**Fig. 2:**
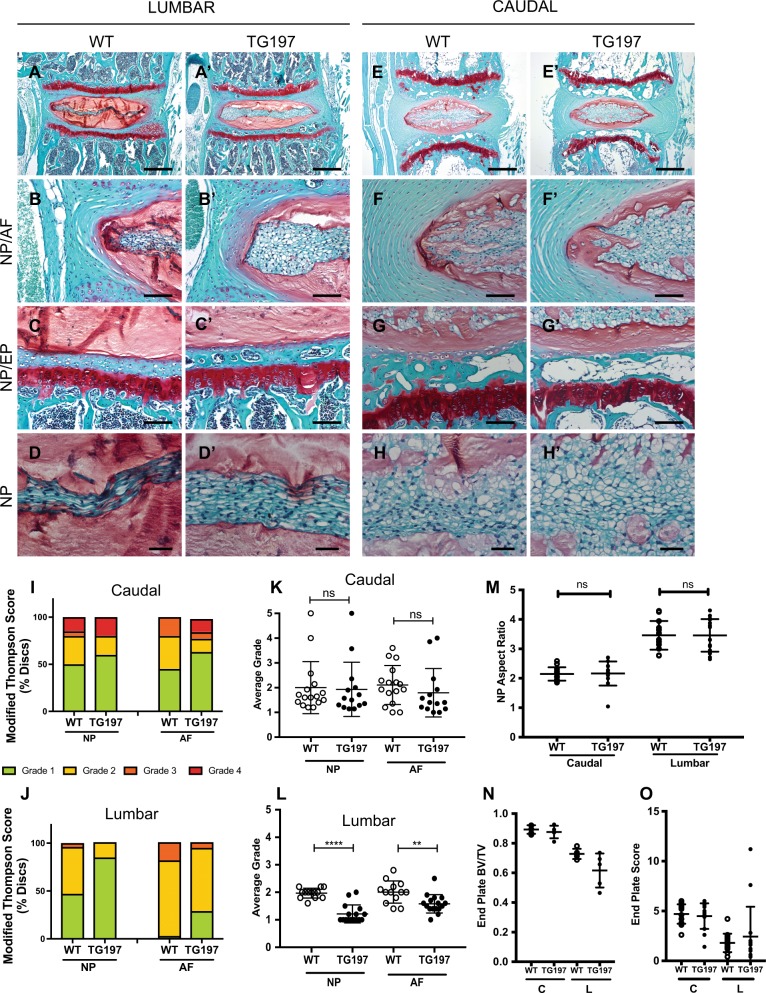
Tg197 discs are healthier or no different than wild-type controls. **a**–**h** Safranin O/Fast Green/Hematoxylin staining of coronal sections of wild-type and Tg197 mouse intervertebral discs. Low magnification images of lumbar Tg197 discs show thickening of the nucleus pulposus cell band. (top row, scale bars = 200 μm; middle rows, scale bars = 50 μm; bottom row, scale bars = 20 μm) **i**, **j** Distribution of histological grades using the modified Thomson scale for **i** caudal and **j** lumbar intervertebral discs. **k**, **l** Average modified Thompson score for **k** caudal and **l** lumbar intervertebral discs. **m** Aspect ratio of caudal and lumbar nucleus pulposus. **n** Bone volume/total volume of endplate in Tg197 and control mice (*n* = 5 mice/genotype). **o** Endplate scoring of caudal and lumbar discs. Data was collected from 3 discs per mouse (*n* = 10 mice/genotype). Significance between average grades was determined using unpaired *t* test. ns = not significant, **p ≤ 0.01, ****p ≤ 0.0001

Tg197 mice have been characterized for their arthritic phenotype. The transgenic animals are smaller than their age-matched WT controls and display signs of arthritic limb malformation and impaired movement^34,36^. Safranin O/Fast green and hematoxylin staining showed that the overall tissue structure of intact caudal and lumbar discs was well preserved and comparable to WT control animals (Fig. 2a–h’). The discs of Tg197 mice were healthy, as evident by the abundant aggrecan-rich extracellular matrix in the NP compartment, vacuolated notochordal NP cells, and well-organized lamellar collagen-rich AF (Fig. 2a–h’). Interestingly, the NP tissue of intact Tg197 discs was more cellular, and the cells appeared larger and vacuolated than the WT controls (Fig. 2a–h’). Unlike the cartilages in other affected joints, the endplates in Tg197 mice showed normal morphology with a layer of hyaline cartilage (CEP) and a subchondral bone plate that was comparable to WT controls (Fig. 2c, c’, g, g’).

To quantitatively assess the histological changes, we used modified Thompson grading and Boos scoring as described previously^7,37^. No apparent differences in the distribution of NP and AF grades were seen for either the lumbar or caudal discs (Fig. 2i, j). To gain further insight into disc health, we compared average NP and AF grades. There was no significant difference between the average grade of caudal NP or AF (Fig. 2k). Interestingly, the average lumbar NP and AF grade in Tg197 mice was significantly lower than the WT control animals (Fig. 2l). Additionally, there was no difference in the NP aspect ratio between the two genotypes (Fig. 2m). Furthermore, micro-computed tomography (μCT) analysis of the bony endplate showed comparable bone volume per total volume (Fig. 2n), and Boos scoring showed no differences in endplate scores between the genotypes (Fig. 2o). Together, these results suggested that the lumbar discs of hTNF-α-overexpressing mice were healthier than the WT controls. Western blot analysis was performed using a specific hTNF-α reactive antibody confirming that hTNF-α was present in the disc of Tg197 mice (Fig. S2A-C). Additionally, immunofluorescence staining of Syndecan 4, a downstream target of TNF-α in the disc, showed increased expression further indicating that there was elevated TNF-α activity in the Tg197 discs (Fig S2D)^38,39^. Together, these results suggested that the Tg197 mice do not evidence signs of early-onset disc degeneration in intact spinal levels.

### The expression of major disc matrix components is unaffected in Tg197 mice

**Fig. 3:**
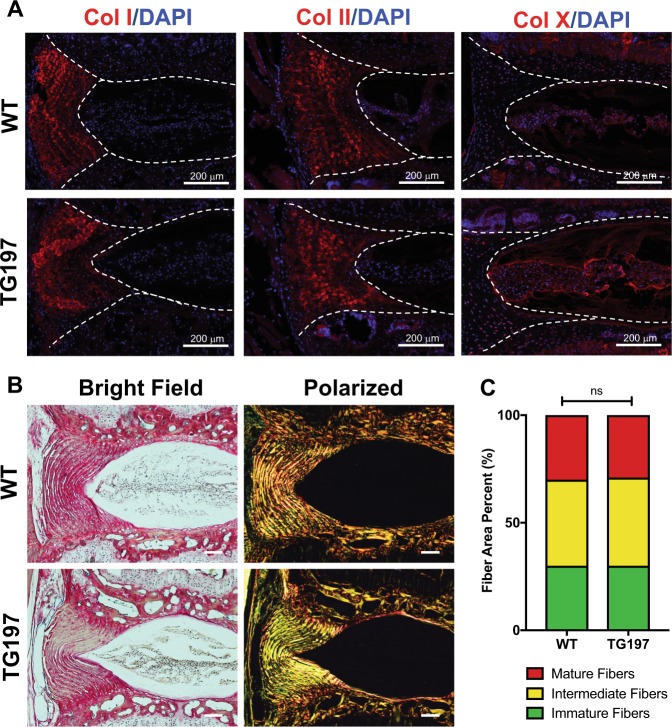
Similarities in histology and immunohistochemistry of Tg197 and wild-type intervertebral discs. **a** Immunofluorescence staining of collagen I, collagen II, and collagen X (red). Collagen I and II showed similar distributions in the annulus fibrosus, while collagen X staining was confined to the nucleus pulposus and slightly elevated in the Tg197 disc. Dotted lines were drawn to distinguish different intervertebral disc compartments. All staining was performed using at least three animals per group. **b** Picrosirius Red staining of 16-week caudal discs showing collagen deposition in the annulus fibrosus and the nucleus pulposus. Collagen fibers visualized under polarized light (right column) show organized lamellae in (scale bar 100 μm). **c** Quantification of the fiber content distribution (*n* = 5). Significance between fiber distribution determined using χ^2^ test. ns = not significant

To assess whether the difference in herniation incidence was due to alterations in the AF matrix composition, we investigated the expression and localization of specific collagens using immunofluorescence staining. Surprisingly, there were no differences in expression levels and localization of collagen I and II between WT and Tg197 discs (Fig. 3a). However, there was a slight increase in collagen X staining in the Tg197 NP compared to WT controls (Fig. 3a). To gain further understanding of the overall collagen architecture and compositional makeup of these discs, Picrosirius Red staining was performed and the collagen birefringence, as an indicator of fiber diameter and maturity, was observed under polarized light^40^. As expected, AF showed strong birefringence, while NP tissue lacked any signal. Quantitative analysis showed that the distribution of fiber sizes in AF was nearly identical between the two genotypes (Fig. 3c). It is important to note that while collagen II is the primary collagen in the NP its concentration in the mouse disc is extremely low compared to that of the AF (Fig. 3b). This is supported by both the immunofluorescence and polarized microscopy, along with previously published studies (Fig. 3a, b)^7,41^.

**Fig. 4:**
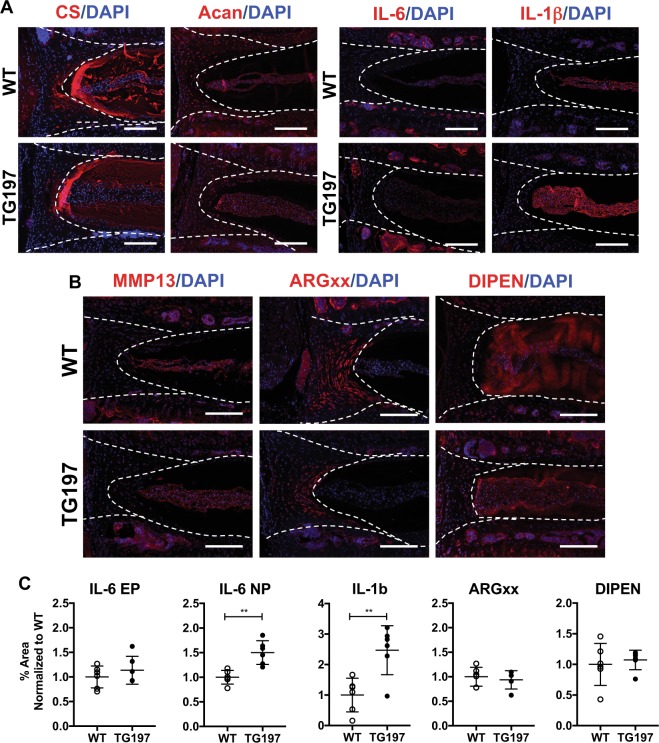
Tg197 discs show altered inflammatory environment without changes in matrix composition. **a** Chondroitin sulfate (CS) and aggrecan (Acan) staining show no differences between WT and Tg197 (*n* = 3 animals/genotype), but IL-6 and IL-1β levels are increased in Tg197 NP (*n* = 6 animals/genotype). **b** MMP-13, ARGxx, and DIPEN show no differences in between the two genotypes. **c** Quantification of staining for IL-6, IL-1β, ARGxx, and DIPEN performed using *n* = 6 mice/genotype. **p ≤ 0.01 Dotted lines in **a**, **b** were drawn to distinguish different intervertebral disc compartments. Scale bar = 200 μm

To gain further insights into matrix composition and inflammatory environment of the Tg197 discs, we stained sections for chondroitin sulfate (CS), aggrecan core protein (Acan), IL-6, IL-1β, MMP13, aggrecanase (ADAMTS-1, -4, and -5)-generated aggrecan N-terminal G1 neoepitope ARG (ARGxx), and MMP-generated N-terminal neoepitope sequence (DIPEN) (Fig. 4a, b). CS, MMP13, Acan, ARGxx, and DIPEN staining showed similar distribution in the NP and AF of WT and Tg197 animals (Fig. 4a, b). While IL-6 staining appeared pronounced within the vasculature of the subchondral bony endplate, quantification showed no significant difference between the genotypes (Fig. 4c). However, both IL-1β and IL-6 staining was significantly higher in Tg197 mice than in WT controls (Fig. 4a–c). Together, these results indicated that, while there were no gross compositional differences between the Tg197 and WT controls, there was some evidence of an altered inflammatory environment in the Tg197 discs.

### Tg197 mice show increased cellularity in NP compartment

**Fig. 5:**
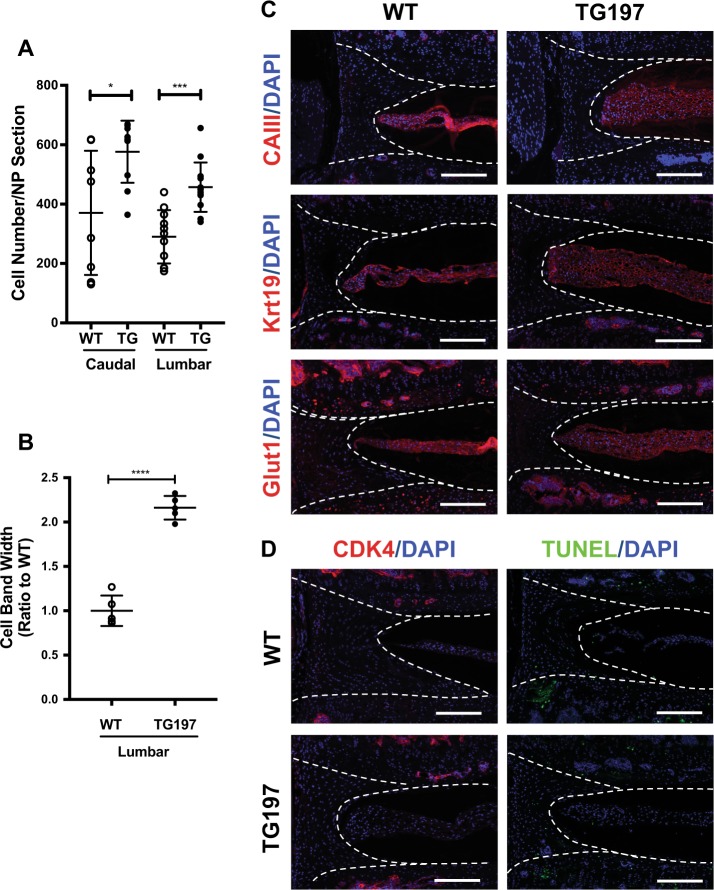
Tg197 lumbar and caudal discs are more cellular than wild-type controls, and the cells in Tg197 mice have comparable nucleus pulposus phenotypes. **a** Both caudal and lumbar Tg197 discs are more cellular than wild-type controls (*n* = 5). **b** Width of lumbar disc cell band in Tg197 mice is more than two-fold larger than wild-type controls (*n* = 5). **c** Immunofluorescence staining of nucleus pulposus markers CAIII, Krt19, and Glut1. The staining between genotype was very similar. **d** Immunofluorescence staining of CDK4 shows no difference in cell proliferation. Tunnel assay of 16-week-old lumbar discs showed no difference in cell death. Dotted lines were drawn to distinguish different intervertebral disc compartments. All staining was performed using at least three animals per genotype. (scale bar = 200 μm) *t* test. *p ≤ 0.05, ***p ≤ 0.001, ****p ≤ 0.0001

Safranin O/Fast green staining clearly showed increase cell number in the NP compartment of Tg197 mice (Fig. 1a). Consequently, we quantified the cellularity of the discs by counting the number of nuclei. The number of NP cells was significantly higher in Tg197 compared to WT animals (Fig. 5a). Not only were there more cells in the lumbar discs of Tg197 mice, but the NP cell band was more than twice as thick as the WT mice, suggesting a possible increase in size of the individual cells (Fig. 5b). To determine whether the NP cells in Tg197 mice are phenotypically similar to WT animals, we measured the expression of known NP cell markers: carbonic anhydrase III (CAIII), keratin 19 (Krt19), and glucose transporter 1 (Glut1)^42,43^. There were no differences in expression and pattern of staining of any of the markers between the WT and Tg197 mice (Fig. 5c). To explore whether the change in cell number was due to active proliferation or cell death, CDK4 and terminal deoxinucleotidyl transferase-mediated dUTP-fluorescein nick end labeling (TUNEL) staining was performed. At this age, there were no CDK4-positive cells in either the Tg197 or control mice indicating a lack of cell proliferation. Additionally, there were few, if any, TUNEL-positive cells in both Tg197 and control mice (Fig. 5d). These results implied that the increased cell number in Tg197 did not arise owing to active cell proliferation or cell death at 12–16 weeks. In summary, these studies showed that disc of Tg197 mice have increased NP cellularity than their age matched WT controls, and these cells maintained their NP phenotype.

### Vertebral bone of Tg197 mice show characteristic thinning

**Fig. 6:**
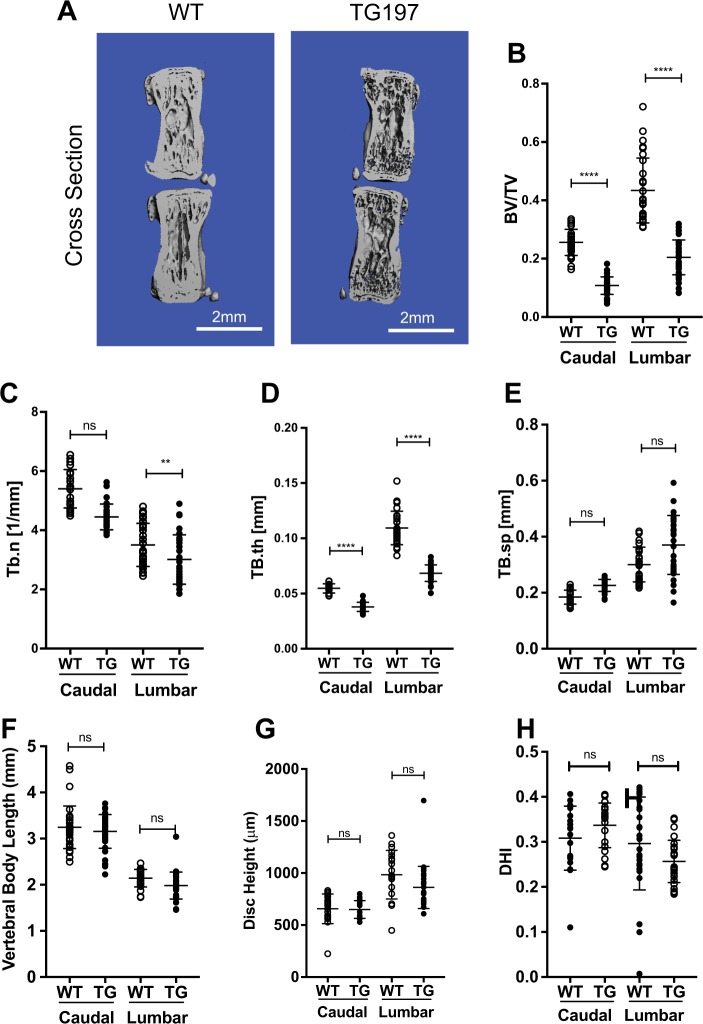
Tg197 vertebrae show characteristic bone erosion and trabecular thinning. **a** Representative μCT scans of caudal motion segments of 16-week-old spines showing gross trabecular thinning. **b**–**h** Bone volume/trabecular volume (BV/TV), trabecular number (Tb.n), trabecular spacing (Tb.s), trabecular thickness (Tb.th), vertebral body length, disc height, and disc height index (DHI) of Tg197 and wild-type mice (mean ± SD) (*n* = 10 per genotype with 3 consecutive vertebrae/animal). Scatter plots show all data points and plotted as mean ± SD. *t* test. ns = not significant, **p ≤ 0.01, ****p ≤ 0.0001

We performed μCT analysis to study vertebral bone properties and measure disc height. μCT studies revealed severe erosion in the lumbar and tail vertebrae (Fig. 6a). There was significant reduction in bone volume per total volume (BV/TV) in the caudal and lumbar vertebrae of Tg197 mice indicating bone erosion (Fig. 6b). Trabecular number (Tb.n.) was also significantly lower in Tg197 lumbar vertebrae (Fig. 6c). There was a significant reduction in trabecular thickness (Tb.th) in both caudal and lumbar levels (Fig. 6d), while trabecular spacing (Tb.sp.) remained unchanged (Fig. 6e). There was a small but significant reduction in vertebral body length in the lumbar but not in the caudal spine of Tg197 mice (Fig. 6f), which was expected owing to the smaller size of the transgenic mice^36^. However, disc height and disc height index (DHI) showed no differences between the WT mice and Tg197 mice (Fig. 6g, h). These results indicated that the systemic inflammation in Tg197 mice severely affected the vertebrae without affecting disc height.

## Discussion

Inflammation, intervertebral disc disease, and LBP are linked. It has been demonstrated that elevated tissue and systemic levels of inflammatory cytokines, in particular TNF-α, characterize symptomatic disc disease^12,25^. However, despite many decades of research, the definitive cause and effect relationship between systemic inflammation and disc degeneration and herniation have not been adequately established in vivo. To explore the role of TNF-α and systemic inflammation in the pathogenesis of intervertebral disc disease, we used the Tg197 mouse, a well-established model of TNF-α-driven systemic inflammation^34^. We report here, for the first time, an in-depth characterization of spinal phenotype in these mutant animals. Tg197 mice develop robust bone and joint inflammation. There was also increased incidence of spontaneous disc herniation in Tg197 mice that involved failure of the AF, the CEP, and tissue at the subchondral bone junction together with a robust immune cell response that was completely lacking in the WT animals. This type of failure is in accord with the clinical picture of disc herniation and the development of acute radicular pain^44^. Surprisingly, despite the occurrence of spontaneous disc herniation, when intact, the Tg197 discs were healthier and evidenced increased NP cellularity compared to their WT controls. Based on these observations, we conclude that elevated systemic TNF-α predisposes animals to spontaneous herniation but is not sufficient to adversely affect the health of the intact discs at this timescale.

Spontaneous annular tears and NP herniation was a notable feature of the spinal phenotype of Tg197 mice, which have not been reported in mice at this early age^45,46^. Gaining insight into the immune response to herniation has important clinical implications; many lumbar disc herniations regress spontaneously not necessitating costly surgery^47^. Current models of AF breach and exposure of NP to the immune system include disrupting the skin, which alone can cause a pain response secondary to an immune response^48^. The immune response to herniation presented herein resembles the response present in human surgical samples; CD68-positive cells are found in the NP space in both Tg197 mice and human surgical samples^11^. Additionally, mast cells were a significant part of the immune response after spontaneous herniation in Tg197 mice and were recently reported in painful human NP tissue^49^. The presence of T cells in the NP extrusion sample further supports the importance of T cell response in disc disease; Kepler et al. found high levels of regulated T cell expressed and secreted/C-C motif ligand 5 in painful intervertebral discs^50^. The pathogenesis of the herniation in Tg197 mice was not clear since there were no discernable differences in the AF extracellular matrix and the organization of the collagen fibrils between the Tg197 and WT mice. However, it is possible that elevated levels of IL-1β and IL-6 within the NP compartment may have reduced the integrity of the already eroded underlying vertebral bone, and this contributed to the increased incidence of failure at the endplate–AF junction at select levels.

A dogma in disc research is the notion that cytokines are a result and driver of disc degeneration. However, intact Tg197 spinal levels, which evidenced severe vertebral inflammation, had discs that were largely unaffected. The histological studies revealed that distribution of NP and AF grades in Tg197 mice were either similar or in the case of the lumbar NP significantly better than the WT animals. While there is a link between bone inflammation, Modic changes, and LBP, the lack of an overt disc phenotype in the Tg197 mice with severe vertebral inflammation disputes the hypothesis that bone inflammation is a driver of disc degeneration. Moreover, considering both the body of research linking elevated cytokine levels to disc degeneration and the severe arthritic phenotype of these animals, the finding that discs in TNF-α transgenic mice maintained their health was surprising. Furthermore, the lower average grades of the lumbar discs in Tg197 mice suggested that the mutant discs were not just functional but in a healthier state than the controls. It is interesting to note that SM/J mice, a recently reported model of early onset spontaneous disc degeneration, are characterized by reduced systemic TNF-α levels when compared with C57BL/6 mice, suggesting a disconnect between TNF-α levels and extent of disc degeneration^7,51^. The healthier NP grades in Tg197 reflected the increased NP cellularity, which is an important criterion in the modified Thompson grading scheme. However, since the NP compartment is avascular and depends primarily on diffusion for both gaseous and nutrition exchange, the long-term implications of increased cellularity in relationship to disc aging are not entirely clear^52^. It is plausible that, with increasing age and sclerosis of endplates, the NP compartment may not be able to support the increased metabolic demands of its cells, and this fact alone may promote degeneration^53–55^. Another surprising finding was that, despite the increased cellularity of the Tg197 mice, the aspect ratio of the NP compartment was similar between the two genotypes. This further confirms the normal phenotype of intact Tg197 discs since changes in disc shape and size are associated with human disc degeneration^56^.

While the most striking effect of constitutive expression of hTNF-α on the intact discs was increased cell number and size, our results indicate that the difference did not arise from increased cell death in WT animals. Instead, the change was likely due to increased NP cell proliferation during early postnatal life in Tg197 mice, as proliferation of NP cells takes place within the first 3 weeks after birth^57^. For this reason, the lack of CDK4 staining in Tg197 and WT mice was not surprising. These findings indicate that the aberrant proliferation of NP cells at these later post-natal time points was not the cause of the observed increase in cell number. Consequently, a conditional approach using disc-specific Cre drivers (e.g., foxA2-Cre or shh-CreERT2) to drive TNF-α overexpression in the NP could discern the age at which TNF-α leads to NP cell proliferation and whether the observed effects are due to local production or diffusion of systemic TNF-α into the disc. Nonetheless, it is important to note that, while late-stage degeneration is associated with reduced cellularity, clusters of proliferating cells exist in the early stages of degeneration^58^. From this viewpoint, the results clearly showed that systemic overexpression of TNF-α affects the disc, whether this effect was transduced through an indirect action of TNF-α or directly through the pro-survival and pro-proliferative actions of TNFRII remains to be seen^59,60^. From a clinical perspective, it is interesting to note that, following discectomy to correct lumbar disc herniation, higher levels of TNFRII protein were correlated with positive pain outcomes; in contrast, raised levels of TNFRI is associated with negative outcomes, suggesting opposing activities of these two TNF-α receptors in human disc disease^61^. Another possibility is that the Tg197 mice were able to block the deleterious effects of hTNF-α by producing endogenous TNF-α inhibitors such as sTNFR or progranulin^62,63^. Recent studies have shown early-onset disc degeneration in progranulin and IL-1ra knockout mice implying the importance of endogenous TNF-α and IL-1β inhibitors in the maintenance of disc health^64,65^. The idea that increased progranulin production protects the Tg197 mice from disc degeneration is also supported by evidence showing that progranulin promotes proliferation of numerous cell types^66–68^.

In summary, while elevated systemic TNF-α is insufficient to promote disc degeneration in intact discs, it predisposes mice to spontaneous herniation. Clinically, herniation and disc degeneration are closely linked: the presence of AF tears leads to significantly worse disc scores^69^. From this perspective, the Tg197 mutant provides a new and exciting animal model to explore many of the closely held assumptions concerning NP cell function and the relationships among systemic inflammation, vertebral inflammation, disc health, and degenerative disc disease.

## Materials and methods

### Mice

All animal care procedures, housing, breading, and the collection of animal tissues, were performed in accordance with a protocol approved by the Institutional Animal Care and Use Committee of Thomas Jefferson University. The Tg197 transgenic mice between the ages of 12 and 16 weeks harbor five copies of hTNF-α transgene previously described by Kollias and colleagues^34^. Both male and female mice were used in these studies.

### Micro-CT analysis

Micro-CT scans (MicroCT40, SCANCO Medical, Switzerland) were performed on the lumbar and caudal levels of Tg197 and WT mice fixed with 4% paraformaldehyde. Ten mice per genotype were used and data was averaged as a mean of 2–3 spinal levels, all levels were plotted. Segments were scanned with an energy of 70 kVp, a current of 114 mA, and a 200-ms integration time producing a resolution of 16 mm^3^ voxel size. Trabecular bone three-dimensional reconstructions of these scans were compiled using Gaussian filter (*σ* = 1.0, support = 1) and converted to binary images with a fixed gray-scale threshold of 200. The data sets were then assessed using the software supplied by the system manufacturer. DHI was calculated by dividing average disc height by height of adjacent vertebral bodies.

### Histological analysis

Spines were decalcified in 20% ethylenediaminetetracetic acid (EDTA) at 4 °C for 15 days before embedding in paraffin and 7-μm mid-coronal sections were prepared. Xylene deparaffinization followed by graded ethanol rehydration preceded all staining protocols. Safranin O/Fast Green/Hematoxylin-stained slides were imaged using Axio Imager 2 microscope, 5×/0.15 N-Achroplan or 10×/0.3 EC Plan-Neofluar objectives, Axiocam 105 color camera, and Zen2TM software (Carl Zeiss). Scoring was performed using a modified Thompson grading scale (S1) by 5–7 blinded observers^70,71^. Endplate scoring was performed by five blinded observers following Boos criterion^37^. Ten mice per genotype with three discs per mouse in both caudal and lumbar levels were used.

### Picrosirius Red^TM^ analysis

Picrosirius Red^TM^ staining visualized localization and quality of the collagen fibrils^40,72^. Stained sections were imaged on a polarizing microscope (Eclipse LV100 POL, Nikon)^7^. Images containing only the AF were used for the subsequent analysis of the surface area occupied by green, yellow, or red pixels. Threshold levels for these three colors remained constant for analysis of all samples.

### Cell number quantification

DAPI (4,6-diamidino-2-phenylindole; Thermo Fisher Scientific, P36934) stained mid-coronal 7-μm sections were used to quantify the cell number in the NP. Three sections per animal (*n* = 10) were used, and the NP area was used for analysis. Using the ImageJ software (NIH), images were converted to 32-bit, then the background was subtracted using rolling = 50, next the images were auto-thresholded, made binary, and then cell number was calculated using the analyze particles function^73^.

### Immunofluorescence microscopy

Mid-coronal 7-μm sections were used for all immunofluorescence studies. Quantitative staining for IL-6, IL-1β, ARGxx, and DIPEN was performed on six animals per genotype, while for NP phenotypic makers and some matrix molecules (CS and Acan) three animals per genotype were used. Sections were de-paraffinized and rehydrated as described above before antigen retrieval. Antigen retrieval was accomplished in an antibody-specific manner by either heated citrate buffer for 20 min or proteinase K for 10 min at room temperature or Chondroitinase ABC for 30 min at 37 °C or TRIS/EDTA. Sections were blocked in 5% normal serum (Thermo Fisher Scientific, 10000 C) in PBS-T (0.4% Triton X-100 in phosphate-buffered saline (PBS)), and incubated with the primary antibody. The primary antibodies used were Aggrecan (1:50, Millipore, AB1031), Collagen I (1:100, Abcam, ab34710), Collagen II (1:400, Fitzgerald, 70R-CR008), Collagen X (1:500, Abcam, ab58632), CA3 (1:150, Santa Cruz, sc-50715), KRT19 (1:3, DSHB, TROMA-III/supernatant), IL-1β (1:100, Novus, NB600–633), CD8 (1:1000, Abcam, ab209775), CD4 (1:1000, Abcam, ab183685), Ly6 (1:500, Abcam, ab2557), CD68 (1:500, Abcam, ab125212), CD31 (1:1000, Abcam 124432), or MMP13 (1:200, Abcam, ab39012) in blocking buffer at 4 °C overnight. For GLUT-1 (1:200, Abcam, ab40084), ARGxx (1:200, Abcam, ab3773), CS (1:300, Abcam, ab11570), DIPEN (1:500, mdbiosciences, 1042002), IL-6 (1:50, Novus, NB600-1131), and tryptase (1:1000, Abcam, ab2378) staining, Mouse on Mouse Kit (Vector laboratories, BMK-2202) was used for blocking and primary antibody incubation. Tissue sections were washed and incubated with the appropriate Alexa Fluor®-594 conjugated secondary antibody (Jackson ImmunoResearch), at a dilution of 1:700 for 1 h at room temperature in dark. The sections were washed again with PBS-T (0.4% Triton X-100 in PBS) and mounted with ProLong® Gold Antifade Mountant with DAPI (Thermo Fisher Scientific, P36934). All mounted slides were allowed to set overnight before visualization with Axio Imager 2 using 5×/0.15 N-Achroplan or 10×/0.3 EC Plan-Neofluar objectives, AxioCam MRm camera, and Zen2TM software (Carl Zeiss). Exposure settings remained constant for all genotypes. Staining percentage of area quantification was performed using the ImageJ software (NIH), thresholds remained constant for each antibody.

### TUNEL assay

TUNEL assay was performed on disc tissue sections using an “In situ cell death detection” Kit (Roche Diagnostic)^7^. Sections were permeabilized with Proteinase K (20 μg/mL) for 15 min at room temperature before the TUNEL assay and imaged as described above.

### Protein extraction and western blotting

Following sacrifice, NP material from lumbar and caudal discs was surgically isolated and homogenized in T-PER^TM^, 1× protease inhibitor mixture (Roche), NaF (5 nM), and Na_3_VO_4_ (200 μm). Proteins were resolved on a 10% sodium dodecyl sulfate-polyacrylamide gel and transferred by electroblotting to polyvinylidene difluoride membranes (Bio-Rad). Ponceau S staining (0.1% (w/v) Ponceau in 5% (v/v) acetic acid) verified equal protein loading. The membranes were washed with TBS and blocked with 5% nonfat dray milk in TBS with Tween 20 and incubated overnight with αhTNF antibody (1:1000, ab6671, rabbit, Abcam) and then with appropriate secondary antibodies for 1 h at room temperature. Immunolabeling was detected using the ECL reagent (Amersham Biosciences). Recombinant hTNF and mTNF (PeproTech) was used to verify antibody specificity.

### Statistics

Ten animals per genotype per time point were used for analysis (*n* = 10), and data are presented as mean ± SD. Differences between genotypes were analyzed using the Student’s *t* test when only two groups presented on graph or one-way analyses of variance with Sidak’s multiple comparison test between groups. Three lumber or tail levels per mouse were combined and averaged for both μCT and histological analysis. At least five independent blinded individuals performed histological grading. Significance between collagen fiber distributions was determined using χ^2^ test. All statistical analyses were done using Prism7 (GraphPad Software). *p* ≤ 0.05 was the threshold for statistical significance.

## Supplementary information

Supplemental Figure 1

Supplemental Figure 2

Supplementary figure legends
