# Supplementary material for: Transgenic mice overexpressing human TNF-α experience early onset spontaneous intervertebral disc herniation in the absence of overt degeneration
Source: Cell Death Dis. 2018 Dec 18;10(1):7. doi: 10.1038/s41419-018-1246-x (PMC6315044; doi:10.1038/s41419-018-1246-x)
Supplement: Supplementary file 3 — Supplementary figure legends [file 41419_2018_1246_MOESM3_ESM.docx]

**Supplemental Figure 1: Control immunofluorescence** **staining of intact Tg197 discs and CD31 staining showing vascular ingrowth.** (A) Safranin O/Fast Green/ Hematoxylin staining of an intact caudal Tg197 disc, and immunofluorescence staining (CD68, a macrophage marker, tryptase, a mast cell marker, and CD4 and CD8, T-cell markers).

**Supplemental Figure 2: Western blot and immunofluorescence showing active hTNF in Tg197 intervertebral discs.** (A) Western blot of different concentrations of recombinant human or mouse TNF-α probed with an anti-human TNF-α antibody showing antibody specificity. (B) Western blot of protein isolated from a wild type and Tg197 mouse discs showing hTNF-α in the mouse discs. (C) Ponceau stain confirming equal protein loading in both wells. (D) Immunofluorescence staining of TNF-α target syndecan 4 confirming TNF-α activity in Tg197 mouse nucleus pulposus. Staining was performed using at least 3 animals per genotype.
